# Symptom management in complex post-traumatic stress disorder (ICD-11), view and experience of patients and their relatives: a mixed methods approach (Research Proposal)

**DOI:** 10.1186/s13104-017-2790-7

**Published:** 2017-09-07

**Authors:** Manuel P. Stadtmann, Andreas Maercker, Jochen Binder, Wilfried Schnepp

**Affiliations:** 1 Department of Health, University of Witten/Herdecke, Alfred-Herrhausen-Straße 50, 58448 Witten, Germany; 2 Department of Psychology, Psychopathology and Clinical Intervention, University of Zürich, Binzmühlestrasse 14/17, 8050 Zurich, Switzerland; 3 Center for Trauma Disorders, Integrierte Psychiatrie Winterthur, Technikumstrasse 81, Winterthur, Switzerland

**Keywords:** Complex posttraumatic stress disorder, CPTSD, ICD-11, Mixed methods research, Pragmatism, Psychiatry, Symptoms, Mental health

## Abstract

**Objective:**

Using the framework of IDC-11, complex post-traumatic stress disorder will be diagnosed using the core criteria of a post-traumatic stress disorder and the presence of at least one symptom from the following three domains: symptoms of emotional dysregulation, negative self-concept, and problems in interpersonal relationships. In the literature, these symptoms are discussed as a common reason for seeking treatment. The symptoms can influence and impair the quality of life. This article describes a mixed methods study with a sequential exploratory design. The aim is to describe specific patient characteristics, levels of symptom burden and perspectives of adult inpatients and to describe the experiences, views and needs of patients’ relatives. The study will also investigate facilitators of and barriers to symptom management. The research will be conducted in four phases. The first phase will assess patients’ symptom burdens. The second phase will use semi-structured interviews to explore attitudes to symptom management and perceptions of patients and their relatives. The third phase will statistically explore hypotheses generated after the qualitative interviews. The fourth phase will mix the quantitative and qualitative results and interpret critically.

**Results:**

The present study will add new results to the growing literature on complex post-traumatic stress disorder. These results could serve as the basis for further research into the development of interventions to improve symptom management.

*Trial registration* Ethical approval has been obtained from the Swiss cantonal ethic commission (Nr. 201500096). This research was also registered to the World Health Organization Clinical Trials Search Portal through the German Clinical Trial Register, Trial DRKS00012268 (21/04/2017)

## Introduction

The beta version of the International Classification of Disease, 11th version (ICD-11), which has been available online since 2015, contains two diagnoses of stress and trauma-related illnesses: posttraumatic stress disorder (PTSD) and complex posttraumatic stress disorder (CPTSD) [1, 2]. In 2018, the World Health Organization (WHO) is expected to approve ICD-11 as the official latest version [1].

Diagnostics for these two disorders vary. For PTSD, the following three symptom domains are proposed: re-experiencing the traumatic event, avoidance of the event and feeling keyed-up, also called hyperarousal [1–5]. The prerequisite is that the affected person develops the symptoms after a stressful situation of exceptional threat or catastrophic extent. This situation can be, for example, torture, abuse of physical or mental nature, a natural catastrophe or accidents. For CPTSD, leading scientists [1–3, 5–7] in compliance with ICD-11 propose diagnosing by using the core PTSD criteria and by the additional presence of at least one symptom of the following three domains: a negative self-concept, problems in interpersonal relationships, symptoms of emotional dysregulation. A negative self-concept comprises low self-esteem, negative beliefs due to traumatic experiences, feelings of guilt and shame. Problems in interpersonal relationships are based on the lack of skills to build and maintain close social relationships. The domain affective dysregulation involves symptoms such as self-harming behavior, dissociation, emotional numbness, anger outbursts, irritability, excessive crying or anhedonia.

The literature currently contains few results on the prevalence of CPTSD [6, 8, 9]. In a study on inpatients by Cloitre et al. [3], 36.1% of the population had CPTSD, 31.8% had PTSD and 32.1% had clinically unremarkable symptoms. Furthermore, the research results suggest that, after child abuse, the probability of developing CPTSD is double that of developing PTSD. A study conducted by Wolf et al. [8] estimated a CPTSD prevalence of 13% for traumatized veterans and a CPTSD prevalence of 0.6% for the US population.

Symptoms in general are discussed in the literature as a common reason for seeking treatment [10–12]. During the trajectory of illness, those affected often experience multiple as well as in themselves competing symptoms [12, 13]. These symptoms can strongly influence and alter life quality and everyday life itself [13, 14]. Not only do they cause distress, but they can also affect social interactions [15]. Dealing with those symptoms and the resulting difficulties in everyday life is often left to the responsibility of patients and their relatives [12, 13].

There is currently little information on the treatment regimen for patients with CPTSD, and no statement can be made on the differential effect of individual trauma focused procedures in CPTSD [3, 5, 6, 16]. It is postulated that the processing of the traumatic experiences is not possible without symptom management for the patients [17, 18]. No study was found that dealt with symptom management in the everyday life of adult patients with CPTSD. Patients’ relatives play a significant role in research on caregivers and symptom management [19–22]. The relatives can be both a facilitator and a barrier to patients’ symptom management [16, 23]. Yet there is little evidence and no corresponding study could be identified on the role of relatives of patients with CPTSD. This study focuses on the collection of variables of adult patients with CPTSD as well as their experiences and the experiences of their relatives in the context of symptom management in everyday life prior to inpatient admission. It could be used to generate more detailed research questions, which could at best result in improved treatment for patients. These results could also serve as a basis for further research into the development of interventions to improve symptom management in everyday life.

## Aims

The goals of this study are as follows:

1. To describe the characteristics and level of symptom burden for adult CPTSD patients through standardized assessment instruments.
2. To explore and to reconstruct the views, perceptions, experiences, facilitations as well as barriers of adult patients with a CPTSD for symptom management in everyday life and the experiences and views of their relatives.
3. To statistically explore hypotheses generated from the qualitative analyses based on the grounded theory [24].
4. To critically interpret the results using the recommendations for mixed methods designs from Creswell et al. [25] and to generate a complementary range of knowledge and results concerning symptom management of adult patients with CPTSD.

## Main text

### Study design

A sequentially exploratory mixed-method design was selected to generate a complementary and broad range of knowledge and results. From a methodological point of view, this research project is guided by the philosophy of pragmatism. In the literature, leading scientists describe pragmatic philosophy as an adequate paradigm in the context of mixed methods [25–27]. One reason for this is that a pragmatic view of the world is based on the compatibility of qualitative and quantitative research methods.

### Setting

This research is being conducted through a partnership between the psychiatric institution Integrierte Psychiatrie Winterthur, Zürcher Unterland (ipw), the University of Witten/Herdecke and the University of Zurich. Ipw is a large, non-profit, community-based organization that provides psychiatric services in the city of Winterthur in the canton of Zürich, Switzerland. It provides a full continuum of clinic and community-based mental health services for individuals with mental health issues. The current study is being conducted at an inpatient mental health ward for psycho-traumatology located in Winterthur, Switzerland. The ward treats approximately 200 patients per year and employs 25 clinicians, such as psychiatrists, psychologists, mental health nurses and other therapists. It provides treatment for a diverse adult population from the German speaking region of Switzerland. Patients can refer themselves or be referred through a psychiatrist or psychologist. The ward has a capacity for 17 patients and has a 24-h shift organization for nurses.

### Participants

Patient participants for the current investigation will comprise a sample collected through consecutive recruitment. All patients referred for regular inpatient treatment offered by the ward and who fulfill the following criteria will be asked if they wish to participate: It must be the patient’s first inpatient treatment on the psycho-traumatology ward. The patient must be between 18 and 60 years old. The diagnosis of a CPTSD based on the ICD-11 Trauma Questionnaire must be fulfilled. The patients should have a good knowledge of German. Also, there must be a relative willing to participate. Exclusion criteria include acute or latent suicidality and a main diagnosis other than CPTSD. Patients who might endanger themselves or others will also be excluded.

### Data collection and analyses

**Fig. 1:**
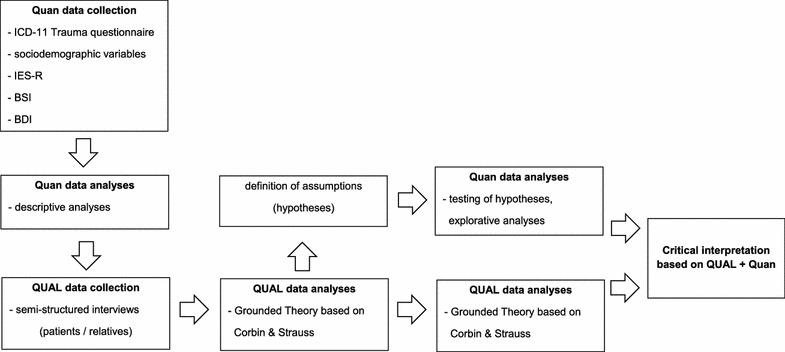
Overview of exploratory sequential mixed methods design

The quantitative part of this research project will begin with data collection through a consecutive sample (see Fig. 1). Based on the ipw statistics from the year 2015, a sample size of about 100 patients can be assumed for the period of data collection. Further sample size calculation is not required as all patients will be included. All participants will receive detailed information during their entry period, and the research intention and aims will be explained. If the patient agrees to participate in the study, informed, written consent will be obtained.

The database for the quantitative study comprises data that will be collected during regular inpatient treatment and is available from the internal clinic database. Timing: T1 = data will be collected during the admission phase by the corresponding practitioner (psychologist or psychiatrist): the sociodemographic information will be collected using a clinical questionnaire, and symptom values will be assessed with the brief symptom inventory (BSI), the beck depression inventory-II (BDI-II) and the impact of events scale-revised (IES-R). Patients who agree to participate in this study will additionally be assessed using the ICD-11 Trauma Questionnaire. This procedure will be completed within the 1st week of inpatient treatment. The ICD-11 Trauma Questionnaire is the new proposed diagnostic questionnaire that is currently under development [3–5]. For this research project, we will use the version translated from English to German by Knefel, Lueger-Schuster and Maercker from 2015. The scale can be used to generate a self-report ICD-11 PTSD or CPTSD diagnosis [28].

The sociodemographic and medical data of the patients are going to be analyzed using the following descriptive statistical methods: absolute and relative frequencies, mean values, minimum and maximum values, percentage, standard deviations, histograms and bar graphs. The Statistical Package for the Social Sciences 24 (SPSS) software will be used for the analyses. If necessary, a MD mass (missing data mass) will be calculated to determine the proportion of missing to existing values. By applying simple or multiple imputation methods, we will attempt to eliminate the issues of the partial high information loss in elimination methods.

In the second phase, the doctoral candidate will be responsible for the qualitative collection of data with semi-structured interviews. Questions are going to be developed based on the results from the first quantitative phase relating to the level of symptom burden. The clinician’s internal code allows the identification of patients by the doctoral candidate, who will ask them for an interview. Based on literature, 30 participants will be needed for data saturation to occur [29–31]. The qualitative sample will be composed of 15 patients and 15 patient relatives. To create the largest possible contrasts between the interview partners, the participants will be chosen through purposive sampling, after the descriptive analyses of the first quantitative phase. A corresponding relative willing to participate and being available for a separate semi-structured interview is a requirement. The qualitative approach serves to capture the experience and the view of patients and their relatives in the context of symptom management in everyday life. The interviews will take place after the inpatient treatment. The data collection will take place between 2017 and 2018 within the psychiatric institution ipw in Winterthur, Switzerland. The semi-structured interviews will take 45 min; an additional 30 min will be available for questions and explanations. The interviews will be recorded and subsequently transcribed verbatim. To comply with data protection, all names will be anonymized. The semi-structured interviews will therefore be analyzed based on grounded theory [24]. After using analytical techniques in open coding, in a second step, the set of categories are reduced and clustered in the phase of axial coding. During the third coding level, the final selection and integration of the categories into a final theory is performed [21, 24, 32]. MAXQDA 12 software will be used for this process. The results of this ongoing analysis will be used to generate hypotheses (Fig. 1).

During the third phase of this study, the hypotheses generated in the second qualitative part will be tested exploratively with the quantitative data already collected in the first phase (Fig. 1). Statistical methods will depend on the hypotheses generated. We assume we possibly will use regression techniques e.g. multiple regressions and methods to assess if the means of two groups are statistically different e.g. t test. Also, here SPSS 24 software will be used for the analyses.

During the fourth and last step, qualitative and quantitative results will be critically interpreted and mixed. Data combination and comparison from the quantitative and the quantitative results will be conducted at the level of data interpretation with a critical social perspective. We will interpret the results using the recommendations for mixed methods designs from Creswell et al. [25]. Priority is given to the qualitative aspects of the study (Fig. 1).

## Discussion

This is the first known study to investigate the symptom management of adult patients with CPTSD. The present trial will add new results to the growing literature on complex posttraumatic stress disorder. This study could be used to generate more detailed research questions, which could improve mental health treatment for patients. For example, this data base could be useful for the development and research of complex interventions to improve symptom management in adult CPTSD patients.

## Limitations

This trial also has limitations that should be considered in evaluating the results. The design as a non-experimental trial has the significant advantage of allowing us to collect data from direct clinical practice. A limitation, however, is the limited comparability. As a study with non-experimental design, there is also no generalizability of the results possible. A further limitation is the data collection in the form of a consecutive sample. Our design did not include a clinical sample other than inpatients in mental health treatment. It is possible that the results will not generalize to other practice settings.

### Trial status

The present study is currently recruiting adult inpatients and their relatives.

## Abbreviations

AR: affect regulation
AV: avoidance
BASEC: Business Administration System for Ethics Committees
BDI-II: beck depression inventory-II
BSI: brief symptom inventory
CPTSD: complex posttraumatic stress disorder
DGPPN: German Association for Psychiatry, Psychotherapy and Psychosomatics
DIPS: Diagnostisches Interview bei psychischen Störungen
DRKS: German Clinical Trial Register
DR: disturbed relationships
DSO: disturbances in self-organization
Fig: figure
ICD-11: International Classification of Disease, 11th version
IES: impact of event scale
IES-R: impact of event scale-revised
ipw: Integrierte Psychiatrie Winterthur
MD mass: missing data mass
NSC: negative self-concept
PTSD: posttraumatic stress disorder
RE: re-experiencing
SPSS: Statistical Package for the Social Sciences
TH: sense of threat
WHO: World Health Organization

## References

[1] Maercker A, Brewin CR, Bryant RA, Cloitre M, Reed GM, van Ommeren M, Humayun A, Jones LM, Kagee A, Llosa AE (2013). Proposals for mental disorders specifically associated with stress in the international classification of diseases-11. Lancet.

[2] Maercker A, Brewin CR, Bryant RA, Cloitre M, Ommeren M, Jones LM, Humayan A, Kagee A, Llosa AE, Rousseau C (2013). Diagnosis and classification of disorders specifically associated with stress: proposals for ICD-11. World Psychiatry.

[3] Cloitre M, Garvert DW, Brewin CR, Bryant RA, Maercker A (2013). Evidence for proposed ICD-11 PTSD and complex PTSD: a latent profile analysis. Eur J Psychotraumatol..

[4] Krammer S, Simmen-Janevska K, Maercker A (2013). Towards ‘complex PTSD’: German translation of the Trauma Symptom Inventory (TSI) for the assessment of complex trauma Sequelae. Psychother Psychosom Med Psychol.

[5] Karatzias T, Shevlin M, Fyvie C, Hyland P, Efthymiadou E, Wilson D, Roberts N, Bisson JI, Brewin CR, Cloitre M (2017). Evidence of distinct profiles of posttraumatic stress disorder (PTSD) and complex posttraumatic stress disorder (CPTSD) based on the new ICD-11 Trauma Questionnaire (ICD-TQ). J Affect Disord.

[6] Hyland P, Murphy J, Shevlin M, Vallières F, McElroy E, Elklit A, Christoffersen M, Cloitre M. Variation in post-traumatic response: the role of trauma type in predicting ICD-11 PTSD and CPTSD symptoms. Soc Psychiatry Psychiatr Epidemiol. 2017;52(6):727–36. 10.1007/s00127-017-1350-8 28194504

[7] Knefel M, Garvert DW, Cloitre M, Lueger-Schuster B (2013). Update to an evaluation of ICD-11 PTSD and complex PTSD criteria in a sample of adult survivors of childhood institutional abuse by Knefel & Lueger-Schuster (2013): a latent profile analysis. Eur J Psychotraumatol.

[8] Wolf EJ, Miller MW, Kilpatrick D, Resnick HS, Badour CL, Marx BP, Keane TM, Rosen RC, Friedman MJ (2015). ICD–11 complex PTSD in US national and veteran samples: prevalence and structural associations with PTSD. Clin Psychol Sci.

[9] Hyland P, Brewin CR, Maercker A (2017). Predictive validity of ICD-11 PTSD as measured by the impact of event scale-revised: a 15-year prospective study of political prisoners. J Trauma Stress.

[10] Lehavot K, O’Hara R, Washington DL, Yano EM, Simpson TL (2015). Posttraumatic stress disorder symptom severity and socioeconomic factors associated with Veterans Health Administration use among women veterans. Women’s health issues.

[11] Cloitre M, Koenen KC, Cohen LR, Han H (2002). Skills training in affective and interpersonal regulation followed by exposure: a phase-based treatment for PTSD related to childhood abuse. J Consult Clin Psychol.

[12] Dodd MJ, Miaskowski C, Lee KA (2004). Occurrence of symptom clusters. Monogr Natl Cancer Inst.

[13] Dodd M, Janson S, Facione N, Faucett J, Froelicher ES, Humphreys J, Lee K, Miaskowski C, Puntillo K, Rankin S (2001). Advancing the science of symptom management. J Adv Nurs.

[14] Engel CC, Litz B, Magruder KM, Harper E, Gore K, Stein N, Yeager D, Liu X, Coe TR (2015). Delivery of self training and education for stressful situations (DESTRESS-PC): a randomized trial of nurse assisted online self-management for PTSD in primary care. Gen Hosp Psychiatry.

[15] Larson P, Viele C, Coleman S, Dibble S, Cebulski C: Comparison of perceived symptoms of patients undergoing bone marrow transplant and the nurses caring for them. In: Oncology nursing forum: 1992; 1992: 81–7 **(discussion 87–88)**. 8421652

[16] Cloitre M, Courtois CA, Charuvastra A, Carapezza R, Stolbach BC, Green BL (2011). Treatment of complex PTSD: results of the ISTSS expert clinician survey on best practices. J Trauma Stress.

[17] Huber M. Trauma und die Folgen: Trauma und Traumabehandlung: Junfermann Verlag GmbH; 2012.

[18] Huber M. Trauma und Traumabehandlung. 2. Wege der Traumabehandlung: Junfermann Verlag GmbH; 2003.

[19] Busch A-K, Schnepp W, Spirig R (2009). Psychosoziale Interventionen für Paare, die mit einer Krebserkrankung leben. Eine Literaturübersicht Pflege.

[20] Mai T, Schnepp W, Höhmann U (2010). Die Lebenssituationen Parkinsonbetroffener und deren Angehörigen im Spiegel der Literatur—ein Überblick. Pflege Die Wissenschaftliche Zeitschrift Fuer Pflegeberufe.

[21] Metzing-Blau S, Schnepp W (2008). Young carers in Germany: to live on as normal as possible–a grounded theory study. BMC Nurs.

[22] Riesner C (2014). Die Rolle pflegender Angehöriger von Menschen mit Demenz in der Bedarfsbestimmung am Beispiel der CarenapD-Studie. Pflege.

[23] Vasterling JJ, Proctor SP, Friedman MJ, Hoge CW, Heeren T, King LA, King DW (2010). PTSD symptom increases in Iraq-deployed soldiers: comparison with nondeployed soldiers and associations with baseline symptoms, deployment experiences, and postdeployment stress. J Trauma Stress.

[24] Strauss AL, Corbin JM, Niewiarra S. Grounded theory: Grundlagen qualitativer sozialforschung: Beltz, Psychologie-Verlag-Union; 1996.

[25] Creswell JW (2013). Research design: qualitative, quantitative, and mixed methods approaches.

[26] Creswell JW, Clark VLP. Designing and conducting mixed methods research. Thousand Oaks, CA: Sage publications; 2007.

[27] Tashakkori A, Teddlie C (2010). Sage handbook of mixed methods in social & behavioral research.

[28] Hecker T, Maercker A (2015). Komplexe posttraumatische Belastungsstörung nach ICD-11. Psychotherapeut.

[29] Francis JJ, Johnston M, Robertson C, Glidewell L, Entwistle V, Eccles MP, Grimshaw JM (2010). What is an adequate sample size? Operationalising data saturation for theory-based interview studies. Psychol Health.

[30] Guest G, Bunce A, Johnson L (2006). How many interviews are enough? An experiment with data saturation and variability. Field Methods.

[31] Mason M. Sample size and saturation in PhD studies using qualitative interviews. In: Forum qualitative Sozialforschung/Forum: qualitative social research. 2010; 2010.

[32] Strauss A, Corbin J (1994). Grounded theory methodology. Handb Qual Res.

